# Guanine and pregnenolone sulfate are associated with incident type 2 diabetes in two independent populations

**DOI:** 10.3389/fendo.2025.1706886

**Published:** 2025-12-03

**Authors:** Maria Barranco-Altirriba, Minerva Granado-Casas, Oscar Yanes, Jordi Capellades, Alexandra Junza, Josep Franch-Nadal, Joan Vendrell, Gemma Llauradó, Sergio Valdés, Eva García-Escobar, Marcelino Bermúdez-López, José Manuel Valdivielso, Victor-Miguel López-Lifante, Cecilia Herrero-Alonso, Mireia Falguera, Maria Belén Vilanova, Ingrid Arteaga, Pere Torán-Monserrat, Alexandre Perera-Lluna, Esmeralda Castelblanco, Didac Mauricio

**Affiliations:** 1 Department of Endocrinology and Nutrition, Hospital de la Santa Creu i Sant Pau, Barcelona, Spain; 2 Bioinformatics and Biomedical Signals Laboratory (B2SLab), Institut de Recerca i Innovació en Salut (IRIS), Universitat Politècnica de Catalunya, Barcelona, Spain; 3 Networking Biomedical Research Centre in the Subject Area of Bioengineering, Biomaterials and Nanomedicine (CIBER-BBN), Madrid, Spain; 4 Institut de Recerca Sant Joan de Déu, Esplugues de Llobregat, Barcelona, Spain; 5 Centro de Investigación Biomédica en red (CIBER) of Diabetes and Associated Metabolic Diseases (CIBERDEM), Instituto de Salud Carlos III (ISCIII), Barcelona, Spain; 6 DAP-Cat Group, Research Support Unit, Institut Universitari d’Investigació en Atenció Primària Jordi Gol, Barcelona, Spain; 7 GESEC Group, Department of Nursing and Physiotherapy, Faculty of Nursing and Physiotherapy, University of Lleida, Lleida, Spain; 8 Healthcare Research Group (GRECS), Institute of Biomedical Research in Lleida (IRBLleida), Lleida, Spain; 9 Department of Electronic Engineering, Institute of Health Research Pere Virgili, Universitat Rovira i Virgili, Tarragona, Spain; 10 Institute of Health Research Pere Virgili (IISPV), Tarragona, Spain; 11 Scientific and Technical Resources Services, Universitat Rovira i Virgili, Tarragona, Spain; 12 Department of Endocrinology and Nutrition, Research Unit, Institut d’Investigació Sanitària Pere Virgili (IISPV) - Hospital Universitari de Tarragona Joan XXIII, Universitat Rovira i Virgili, Tarragona, Spain; 13 Department of Endocrinology and Nutrition, Hospital del Mar, Hospital del Mar Medical Research Institute (IMIM), Barcelona, Spain; 14 UGC Endocrinología y Nutrición, Hospital Regional Universitario de Málaga, IBIMA-Plataforma BIONAND, Málaga, Spain; 15 Experimental Medicine Department, University of Lleida, Lleida, Spain; 16 Vascular and Renal Translational Research Group, Institut de Recerca Biomédica IRBLleida, Lleida, Spain; 17 Faculty of Medicine, Universitat Autònoma de Barcelona (UAB), Barcelona, Spain; 18 Unitat de Suport a la Recerca Metropolitana Nord, Institut Universitari d’Investigació en Atenció Primària Jordi Gol (IDIAP Jordi Gol), Mataró, Spain; 19 Primary Healthcare Palau-Solità i Plegamans, Gerència d’Àmbit d’Atenció Primària Metropolitana Nord, Institut Català de la Salut, Barcelona, Spain; 20 Institute for Biomedical Research Dr. Pifarré Foundation IRB Lleida, University of Lleida and Primary Health Care Centre Tàrrega, Gerència d’Atenció Primaria, Institut Català de la Salut, Lleida, Spain; 21 Primary Health Care Centre Igualada Nord, Consorci Sanitari de l’Anoia, Igualada, Spain; 22 Primary Healthcare Center Vall del Tenes, Gerència d’Àmbit d’Atenció Primària Metropolitana Nord, Institut Català de la Salut, Barcelona, Spain; 23 Department of Medicine, Faculty of Medicine, Universitat de Girona, Girona, Spain; 24 Multidisciplinary Research Group in Health and Society (GREMSAS), Institut Universitari d’Investigació en Atenció Primària Jordi Gol (IDIAPJGol), Barcelona, Spain; 25 Division of Endocrinology, Metabolism and Lipid Research, Department of Medicine, Washington University School of Medicine, St. Louis, MO, United States; 26 Institut d’Investigació Biomèdica Sant Pau (IR-Sant Pau), Barcelona, Spain; 27 Faculty of Medicine, University of Vic - Central University of Catalonia, Vic, Spain

**Keywords:** incident type 2 diabetes, liquid chromatography, mass spectrometry, untargeted metabolomics, targeted metabolomics

## Abstract

**Background:**

Type 2 diabetes (T2D) is increasing its burden worldwide; therefore, research focused on its prediction and prevention is essential.

**Methods:**

We performed an untargeted metabolomics analysis using ultra high-performance liquid chromatography-mass spectrometry to discover metabolic biomarkers and biological pathways associated with incident T2D with a nested case–control design, followed by validation with targeted metabolomics in an independent cohort. In the discovery phase, plasma samples from 352 subjects (209 controls and 143 incident cases) were analyzed, collected with a mean (standard deviation) of 7.40 (0.76) years before they acquired the condition. Using this discovery phase cohort, six metabolites were identified using standards and were subsequently quantified in an independent validation phase cohort of 2,044 subjects (167 incident cases). Additionally, pathway enrichment was conducted in the discovery cohort.

**Results:**

Guanine, ecgonine, adenine, pregnenolone sulfate, phenyl sulfate, and citrulline were significantly associated with incident T2D in at least one of the analyses performed in the discovery phase. Among these, guanine, pregnenolone sulfate, and citrulline maintained their significant associations with incident T2D in the validation cohort. Additionally, several pathways were significantly altered, with nucleotide metabolism and ABC transporter pathways among the most consistently affected.

**Conclusion:**

We identified significant associations of guanine, pregnenolone sulfate, and citrulline with incident T2D.

## Highlights

- Research focused on type 2 diabetes prediction and prevention is essential.
- We investigated metabolites associated with incident type 2 diabetes.
- Guanine, pregnenolone sulfate, and citrulline are potential metabolic biomarkers.
- Nucleotide metabolism and ABC transporter pathways were consistently altered.

## Introduction

Diabetes mellitus (DM), a leading cause of global mortality and disability, is characterized by diverse metabolic disturbances and is increasing at an alarming rate, with the majority of cases being type 2 diabetes (T2D) (1). Research on its causes and prevention is therefore essential.

Metabolomics involves the comprehensive characterization of small molecules in biological systems, and numerous studies have used this approach to investigate T2D risk (2). In particular, associations between T2D risk and metabolites from different classes, such as acylcarnitines, amino acids, and ceramides, have been identified using both targeted and untargeted metabolomics approaches (3–6).

Untargeted metabolomics, while advantageous for discovering novel biomarkers without a prior hypothesis, also faces challenges in identifying condition-associated features and interpreting results globally. Often, only a small subset of significant features can be confidently identified for pathway enrichment, limiting biological insights. To address this, the mummichog algorithm predicts altered pathways directly from unidentified features using putative annotations (7). Concurrently, annotation algorithms have advanced through computational strategies aimed at improving metabolite identification, such as mWISE (8). Annotation refers to the process of assigning a putative identity to each detected feature, generally assuming low confidence (9).

Over the years, several comprehensive metabolome-wide investigations have been conducted, some of which suggest moderate improvements in predicting incident T2D when metabolites are included in models alongside traditional risk factors. However, further optimization and validation are required before such models can be integrated into clinical practice (2).

In this study, we conducted an untargeted metabolomics analysis using ultra high-performance liquid chromatography-mass spectrometry (LC-MS) to extensively profile the serum metabolome of individuals who developed incident T2D. We identified potential biomarkers associated with T2D onset and validated these findings in an independent cohort. We examined associations between baseline metabolite levels and incident diabetes in the full study population, in participants with prediabetes at baseline, and after excluding those who developed prediabetes during follow-up. We also assessed how metabolic features related to glycemic status (normoglycemia, prediabetes, and T2D) at follow-up, as well as transitions between these states. Finally, we identified significantly altered pathways in the discovery cohort.

## Methods

This study was approved by the local ethics committee of the Hospital de la Santa Creu i Sant Pau (ref. 19/098) and IDIAP Jordi Gol (ref. 19/167-P), following the principles of the Declaration of Helsinki. All participants provided written informed consent.

### Study populations

The untargeted metabolomics analysis was initially performed in a “discovery phase” cohort, and the findings were further analyzed and validated in an independent cohort (i.e., the “validation phase” cohort).

### Discovery phase cohort

A nested case–control design was used in the discovery phase of the study using the di@bet.es cohort (10). The di@bet.es is a population-based cohort study with a follow-up of 7.5 (0.6) years. Additional information can be found in the supporting information.

All available incident T2D cases were considered, and two controls for each case were selected by matching time-to-event, sex, age, and body mass index (BMI) at baseline using the propensity score (PS) methodology (ratio 1:2, caliper tolerance level = 0.25, method = nearest neighbor) (11). After matching, a total of 143 cases remained. Samples with missing values in some of the variables used in the models were removed. In untargeted metabolomics studies, a formal *a priori* sample size calculation is typically not performed, as the primary aim is to perform an exploratory profiling rather than testing the hypothesis. However, a sample size of at least 100 individuals is generally considered adequate to ensure sufficient statistical power and coverage of metabolic variability.

Incident diabetes was defined as having a fasting serum glucose level of ≥7 mmol/L, a 2-h post-load serum glucose of ≥11 mmol/L, a glycated hemoglobin (HbA1c) level of ≥6.5% (47.54 mmol/mol Hb), or the use of glucose-lowering medication(s) during the follow-up examination. “Known diabetes” was determined when the participant reported a diabetes diagnosis and/or treatment with diabetes medication(s).

### Validation phase cohort

The validation phase cohort included subjects from four different prospective cohorts: designated as ILERVAS (12, 13), Mollerussa (14), FIBROSCAN (15), and ARTPER (16) studies. After applying the exclusion criteria from an initial pool of 5,777 subjects with available samples and data (**Supplementary Figure S1**), 1,060 individuals with prediabetes and 1,060 subjects with normoglycemia were selected. Assuming an annual diabetes incidence rate of 5% in the prediabetic population, at least 196 individuals (see **Equation 1 in the Supporting Information**) were estimated to become diabetic after a 4-year follow-up period.

The subjects were matched by sex, age, cohort, BMI, dyslipidemia (DLP), and hypertension (HT) using the PS methodology. The SIDIAP database (17) was used to determine incident diabetes at follow-up. Some samples were excluded due to technical problems or missing values in some variables. A brief description of each cohort is available in the Supporting Information.

In all cases, blood samples were collected in the fasting state. Diabetes at follow-up was defined as meeting at least one of the following criteria: fasting serum glucose level of ≥126 mg/dL, HbA1c level of ≥6.5%, use of glucose-lowering medication, or having a registered diagnosis of T2D.

### Untargeted analysis

#### Metabolite extraction

For the extraction of metabolites, 25 µL of plasma was thawed at 4°C followed by a brief vortex mix. Proteins were precipitated by the addition of 200 μL cold acetonitrile/methanol/water (5:4:1, v:v:v) followed by 10 s of vortex mixing. The samples were then kept on ice for 30 min. After centrifugation (10 min, 12,000 rpm at 4°C), the supernatant was transferred to an LC autosampler vial.

#### LC-MS settings

LC/MS was performed using a Thermo Scientific Vanquish Horizon UHPLC system interfaced with a Thermo Scientific Orbitrap ID-X Tribrid Mass Spectrometer (Waltham, MA). Hydrophilic interaction liquid chromatography (HILIC) was performed using an ACQUITY UPLC BEH HILIC column (Waters) with the following specifications: 150 mm × 2.1 mm, 1.7 μm. The mobile-phase solvents were A = 50 mM ammonium acetate and B = acetonitrile. A linear gradient was applied at a flow rate of 400 μL/min: 0–2 min: 95% B, 2–6 min: decreased to 50% B, 6–7 min: isocratic of 50% B, 7-7.2 raised to 95% B, 7.2-10.5 min re-equilibration column at 95% B.

Data collection settings included spray voltage, 3.5 kV (positive mode) and −2.8 kV (negative mode); sheath gas, 50; auxiliary gas, 10; sweep gas, 1; ion transfer tube temperature, 300°C; vaporizer temperature, 200°C; mass range, 70-1,000 Da; RF lens, 60%; resolution, 120,000 (MS1); AGC target, 2e5; maximum injection time, 200 ms (MS1), isolation window, 1 Da. The injection volume was 5 μL.

Quality control (QC) samples consisting of pooled samples were injected at the beginning and periodically through the workflow.

An LC-MS/MS method using a Data-Dependent Acquisition (DDA) dynamic Inclusion List (IL) was used for metabolite identification. The instrument settings were similar to those used in the untargeted experiments, with the following differences: resolution, 15,000; AGC target, 5e4; HCD fragmentation spectra acquired using a stepped method combining a mix of energies (10%, 20%, 30%, 40%); and the injection time that was defined by HERMES (18) exported IL in csv format.

#### Metabolite identification by MS/MS

Preliminary metabolite identification was performed using two strategies: cosine spectral matching within RHermes using an in-house database containing MS/MS spectra from MassBankEU, MoNA, HMBD, Riken, and NIST20 databases; and matching against the mzCloud database using MassFrontier version 8.0 SR1 (Thermo Scientific). Spectral hits with high similarity scores (>0.8) were manually reviewed to confirm metabolite identifications. Matches were restricted to compounds with formulas present in the HERMES formula database. Furthermore, the identities of significant metabolites were confirmed through manual revision of spectral matches and annotation of mismatched fragments via manual spectral elucidation.

#### Data processing

LC–MS data were processed using the HERMES R package (18) for MS1 profiling, quantification, and IL generation for metabolite identification. The database used contained 22,314 unique molecular formulas from ChEBI and HMDB. Quantification was performed using the xcms (19–21) pipeline, which included peak-picking, alignment, grouping, and gap filling. xcms-features were assigned to RHermes Spectral Object of Interest (SOI) using an in-house script.

### Targeted analysis

#### Metabolite extraction

Metabolites were extracted from 25 µL of plasma by adding 200 µL of cold acetonitrile:methanol:water (5:4:1/v:v:v) containing 300 parts per billion (ppb) of d3-Leucine and 200 ppb of aminoterephthalic acid as internal standards. The samples were vortexed and kept on ice for 30 min to allow for protein precipitation. Following this, the samples were centrifuged at 4°C for 10 min at 12,000 rpm, and the supernatant was transferred to an LC-MS vial.

#### LC-MS settings

LC/MS was performed using a Thermo Scientific Vanquish Horizon UHPLC system interfaced with a Thermo Scientific Orbitrap ID-X Tribrid Mass Spectrometer (Waltham, MA). Metabolite separation was achieved using an ACQUITY UPLC BEH HILIC (2.1 × 150mm, 1.7µm) column (Waters).

The chromatographic method was optimized to reduce the overall runtime while maintaining effective separation of the targeted metabolites. Mobile phase A consisted of 50 mM ammonium acetate in water, whereas mobile phase B was acetonitrile. The gradient used for separation was as follows: 0–2 min, isocratic 96% B; 2–5.5 min, decreased to 50% B; 5.5-6.3 min, isocratic 50% B; 6.3-6.5 min, increased to 96% B; 6.5–9 min, re-equilibration column 96% B. The flow was 0.5 mL/min and the injection volume was 5 µL.

#### Targeted method settings

Samples were analyzed using a targeted Selected Ion Monitoring (SIM) mode. Positive polarity was applied for guanine, whereas negative polarity was used for the remaining metabolites (**Supplementary Table S1**). The MS parameters used were as follows: isolation mode, ion trap; isolation window (*m/z*), 2; positive spray voltage, 3,500 V; negative spray voltage, 2,800 V; sheath gas, 50; auxiliary gas, 10; ion transfer tube temperature, 300°C; vaporizer temperature, 300°C; Orbitrap resolution, 60,000; RF Lens (%), 60; AGC target, 2e5; maximum injection time, Auto. Data were acquired in four batches. The first two batches contained two sub-batches.

### Statistical analysis—discovery

Prior to statistical analysis, features were filtered using the 80% rule (22), missing values were imputed as half the minimum value for each feature, and data were log-transformed. Outliers were detected by applying the boxplot.stats function in R to each feature, flagging samples that fell beyond the whisker extremes. Samples classified as outliers in more than 20% of the features were excluded.

After these steps, technical bias was addressed using three parallel approaches: 1) Quality Control-Robust Spline Correction (QC-RSC) using the pmp R package (23), 2) common principal component analysis (CPCA) introduced by Fernández-Albert et al. (24), and 3) retaining non-corrected data. For the CPCA approach, the number of common principal components (CPCs) used for correction was selected by calculating the Euclidean distance between the corrected QCs and the QCs centroid after correcting for up to four CPCs. The CPC count that minimized this distance was chosen.

Following each correction method, the data were scaled and centered. Finally, a coefficient of variation (CV)-based filter was applied to remove features with a higher CV in the QC samples than in the individual samples. The entire workflow is summarized in **Supplementary Figure S2**.

After processing, the clinical data of the participants were summarized as mean (standard deviation) for continuous variables, and as frequency (percentage) for categorical data, using the compareGroups R package (25).

Statistical analysis involved multiple strategies, each preceded by a partial least squares discriminant analysis (PLS-DA). Features with variable importance in projection (VIP) scores higher than 1 were retained for further analysis. To ensure that the identified metabolites were independent of traditional risk factors, models included sex, age, BMI, hypertension, insulin resistance (HOMA-IR), smoking, time to event, glucose, and family history of diabetes. Four outcomes were analyzed: (1) T2D development as a binary variable; (2) glycemia status at follow-up (0 for normoglycemia, 1 for prediabetes, 2 for T2D); (3) glycemia transition in baseline normoglycemic individuals (to normoglycemia, prediabetes, or T2D); and (4) transition in those with baseline prediabetes (to normoglycemia, prediabetes, or T2D). Additionally, different subsets of subjects were considered. **Supplementary Table S2** shows the different analyses implemented. The false discovery rate in each analysis was controlled using the qvalue R package (26), and a corrected p-value lower than 0.05 was considered as significant.

An additional interpretation step was performed using mWISE (8) for LC-MS feature annotation and FELLA (27) for pathway enrichment analysis, prioritizing for enrichment those analyses with the greatest number of significant features. Data were first annotated with mWISE, selecting the top three candidates per feature, which were then used for pathway enrichment in FELLA using both diffusion and hypergeometric methods. Only pathways with at least three hit metabolites and statistically significant enrichment were considered for visualization and discussion. In parallel, to explore baseline population characteristics, features associated with insulin resistance were analyzed using the same interpretation workflow. The mWISE algorithm was applied using the configuration that showed the best performance in the original study (8), namely, the binary unique input, the raw score, and the FELLA-type graph. All other parameters were kept at their default values, both in this step and in the subsequent pathway enrichment analysis (8).

### Statistical analysis—validation

Prior to statistical analysis, data were cleaned and corrected. First, the 80% rule was applied one batch at a time, in such a way that metabolites with more than 20% of the samples without signal in at least one of the sub-batches were removed. Then, imputation by the half of the minimum value of the metabolite was conducted with the remaining missing values, and metabolite intensities were log-transformed and scaled. A principal component analysis (PCA) plot was used to qualitatively observe the presence of technical bias in the QC samples. Then, ComBat function from the sva R package (28) was used to correct the batch effect, considering all sub-batches. The distribution of the six validated metabolites was visualized using histograms.

The clinical data were again summarized using the compareGroups R package. Afterward, the same models applied to the discovery data were implemented with the validation data, but using slightly different confounding factors. In this case, sex, age, BMI, hypertension, smoking habit, time-to-event, and prediabetes were used.

Finally, the prediction ability of the validated metabolites was evaluated using stratified K-fold (K = 5) cross-validation to account for data unbalance. Weighted logistic regression was used to predict the development of T2D using 1) clinical variables (model M1), 2) clinical variables and all metabolites (model M2), and 3) clinical variables and the significant metabolites in validation (model M3). The three models were applied to three different subsets of the data: 1) all the subjects, 2) subjects with prediabetes at baseline, and 3) subjects without prediabetes at follow-up. In all cases, the clinical variables used were sex, age, BMI, hypertension, and smoking habit. In the first and third subsets of data, prediabetes at baseline was also included.

The code used for the statistical analysis is available at https://github.com/m-baralt/metabolomics_incident_diabetes.

## Results

### Data preparation

In the discovery phase, the number of features retained varied across correction types (**Supplementary Table S3**), as the CV-based filter was applied after the QC-based correction, impacting its outcome. PCA plots (**Supplementary Figure S3**) reveal clear technical biases in the uncorrected data, which are substantially reduced following CPCA and QC-RSC correction. Specific PCA plots for the CPCA correction (**Supplementary Figures S4**, **S5**) indicate that three and four common components were optimal for the positive and negative ionization modes, respectively.

In the validation phase, all metabolites passed filtering, suggesting good data quality. No QC-based corrections were needed (**Supplementary Figure S6**). Instead, batch effects were identified by examining PCA plots and subsequently corrected (**Supplementary Figure S7**). After correction, the metabolite distributions (**Supplementary Figure S8**) approximated normality, confirming successful normalization.

### Baseline characteristics

In the discovery cohort, insulin resistance, elevated glucose levels, and a family history of diabetes were positively associated with incident T2D. In contrast, the validation cohort showed positive associations between incident T2D and male sex, hypertension, prediabetes, and higher BMI (**Table 1**).

**Table 1:** Baseline characteristics of subjects without diabetes and with T2D at follow-up in the discovery and validation cohorts.

Clinical variables	Discovery			Validation		
	Without T2D (N = 202)	With T2D (N = 136)	P-value	Without T2D (N = 1877)	With T2D (N = 167)	P-value
Sex (men)	92 (45.5%)	60 (44.1%)	0.883	826 (44.0%)	93 (55.7%)	0.005
Age (years)	57.3 (12.4)	56.4 (11.9)	0.498	58.5 (6.72)	59.2 (6.48)	0.181
BMI (kg/m2)	30.4 (4.62)	30.8 (4.92)	0.444	29.6 (4.81)	32.0 (5.37)	<0.001
HT (yes)	107 (53.0%)	81 (59.6%)	0.278	816 (43.5%)	92 (55.1%)	0.005
HOMA-IR	2.34 (1.50)	3.04 (1.83)	<0.001	–	–	–
Smoking:						
Non-smoker	117 (57.9%)	64 (47.1)	0.081	833 (44.4%)	63 (37.7%)	0.084
Former smoker	52 (25.7%)	38 (27.9%)		577 (30.7%)	65 (38.9%)	
Current smoker	33 (16.3%)	34 (25.0%)		467 (24.9%)	39 (23.4%)	
Time-to-event (years)	7.39 (0.67)	7.41 (0.87)	0.786	4.53 (1.70)	3.14 (1.90)	<0.001
Glucose (mg/dL)	95.2 (10.6)	103 (13.2)	<0.001	–	–	–
Family history DM (yes)	102 (50.5%)	87 (64.0%)	0.02	–	–	–
Prediabetes (yes)	–	–	–	872 (46.5%)	133 (79.6%)	<0.001

Data are mean (standard deviation) for continuous variables and number (%) for categorical variables. For continuous variables, the p-values were obtained using a Student’s t-test and for categorical variables, a chi-squared test. T2D, type 2 diabetes mellitus; BMI, body mass index; HT, hypertension; HOMA-IR, Homeostatic Model Assessment for Insulin Resistance; DM, diabetes mellitus.

### Analysis in the discovery phase

**Supplementary Table S4** summarizes the number of significant metabolite features identified in each analysis, comparing different technical bias correction methods across positive and negative ionization modes. Among these significant features, seven were confidently identified using tandem mass spectrometry and standards (**Table 2**), representing six unique metabolites: guanine, ecgonine, adenine, pregnenolone sulfate, phenyl sulfate, and citrulline.

**Table 2:** Statistical results for six identified metabolites.

Compound	Mass-to-charge ratio	Retention time	Analysis	q-value	Correction	Ionization
Guanine	152.05669	282.90	R1	0.046	QC-RSC	Positive
Guanine	152.05669	271.25	R3; R1	0.045; 0.046	CPCA; QC-RSC	Positive
Ecgonine	186.11274	358.16	R1	0.046	QC-RSC	Positive
Adenine	134.04722	254.21	R1|R3; R3|R4	[0.040, 0.048]; [0.026, 0.031]	CPCA; None	Negative
Pregnenolone sulfate	395.18977	39.70	R1	0.050	CPCA	Negative
Phenyl sulfate	172.9914	63.03	R4	0.033	None	Negative
Citrulline	174.08842	363.89	R1|R3; R4	[0.044, 0.048]; 0.045	CPCA; None	Negative

**Figure 1:**
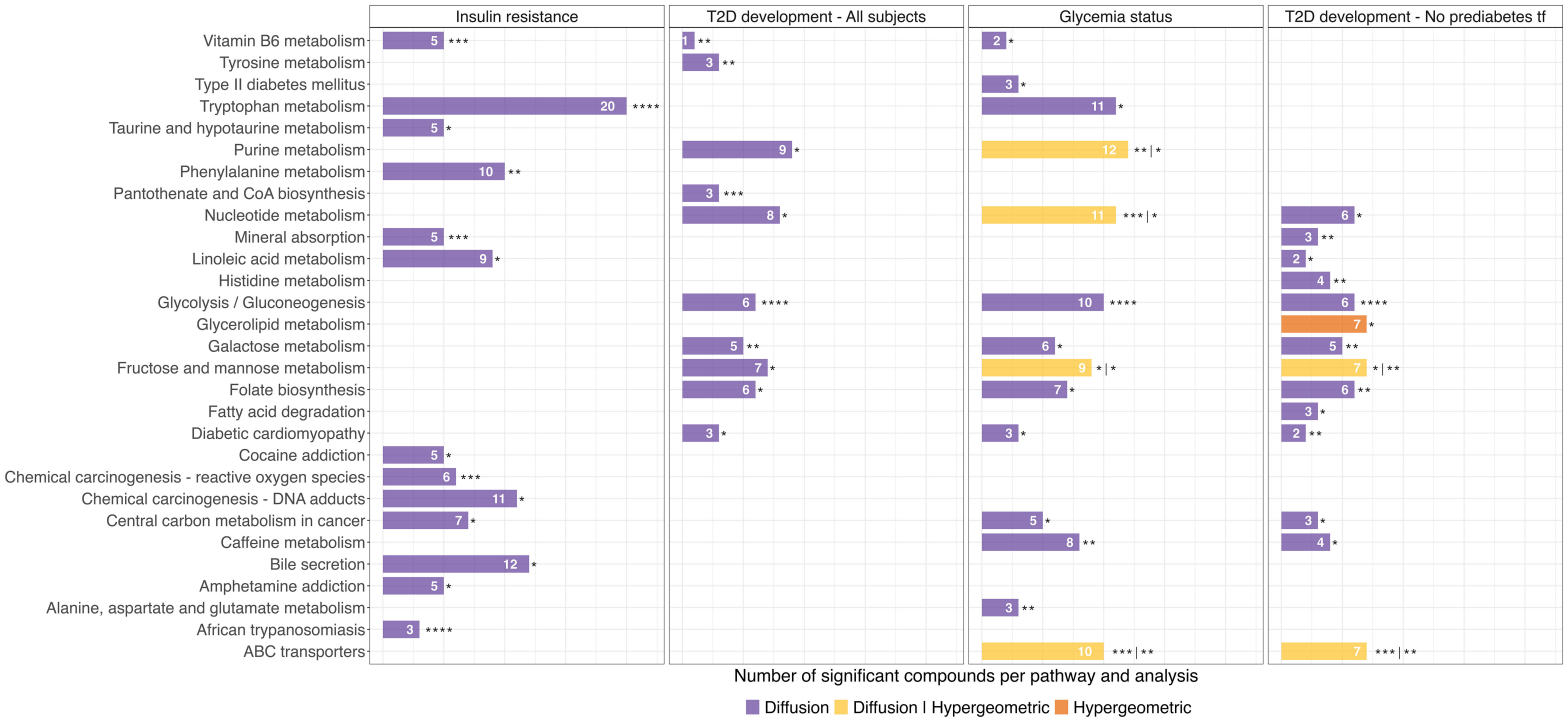
Significant pathways identified using diffusion and hypergeometric approaches in FELLA. The number of significant metabolites within each pathway is displayed in white. Statistical significance is indicated by asterisks: p-value (p) ≤ 0.05 (*), p ≤ 0.01 (**), p ≤ 0.001 (***), p ≤ 0.0001 (****). For pathways identified as significant by both approaches, the significance of each test is separated by a vertical bar.

Pathway enrichment analysis with FELLA (**Figure 1**) highlights key metabolic pathways altered in individuals with incident T2D.

### Validation phase

**Figure 2:**
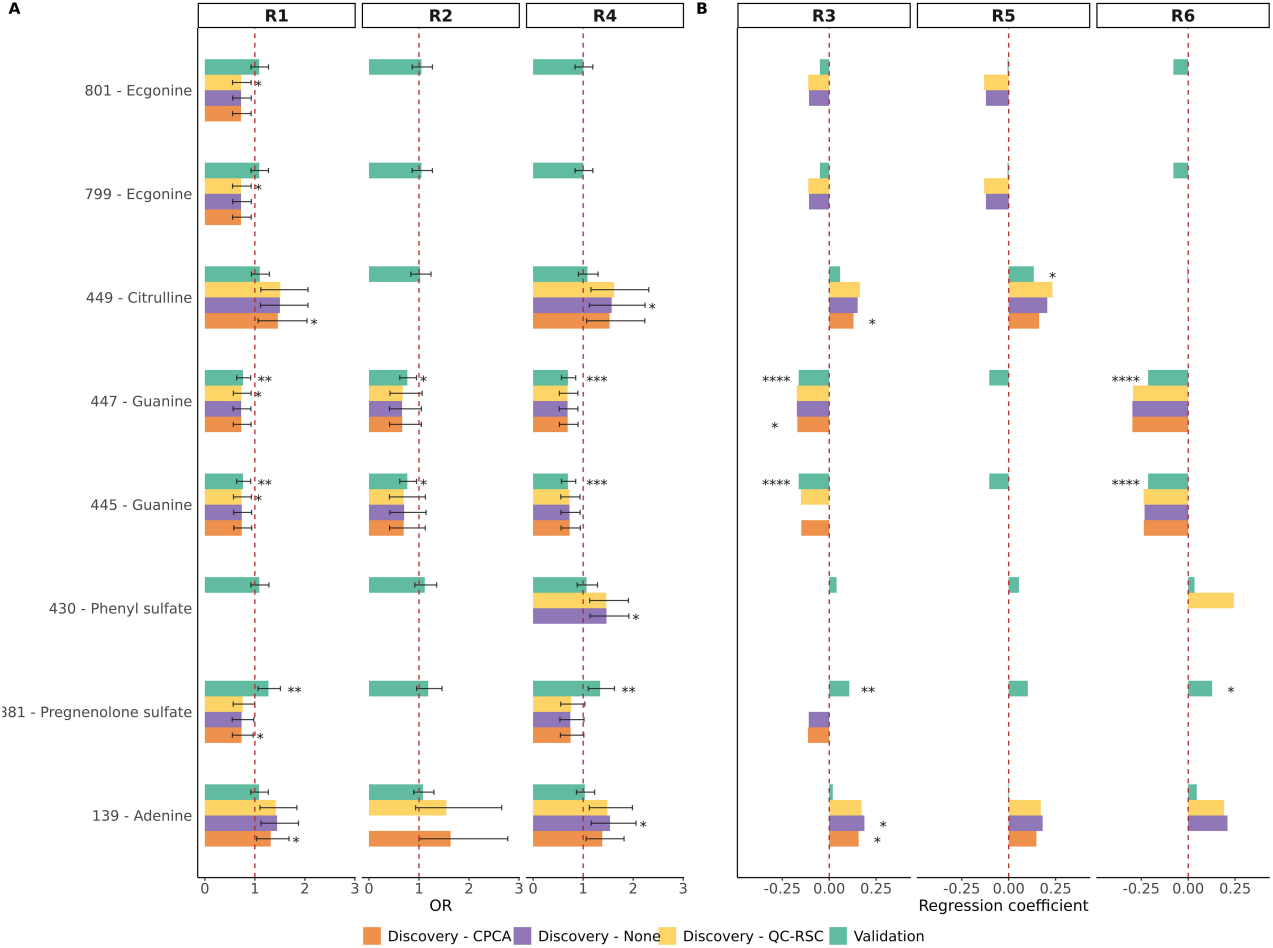
Association of the six identified and validated metabolites in the discovery and validation phases. Plot **(A)** shows the odd ratios for the logistic regression models and **(B)** the regression coefficients for the linear regression models. Missing bars indicate metabolites that, in the discovery phase of a specific analysis, did not have a variable importance in projection (VIP) score greater than one in the partial least squares discriminant analysis (PLS-DA). Statistical significance is indicated using asterisks: p-value (p) < 0.05 (*), p < 0.01 (**), p < 0.001 (***), p < 0.0001 (****). R1 analyzes T2D development using all subjects; R2 analyzes T2D development using individuals with prediabetes at baseline; R3 analyzes glycemia status using all individuals; R4 analyzes T2D development discarding individuals without prediabetes at the end; R5 analyzes glycemia transition using individuals with normoglycemia at baseline and R6 analyzes glycemia transition using individuals with prediabetes at baseline (**Supplementary Table S2**).

Guanine, pregnenolone sulfate, and citrulline showed significant associations with incident T2D or glycemia in both cohorts (**Table 3**). **Figure 2** summarizes key findings from both phases, highlighting the reproducibility and potential relevance of these metabolites.

**Table 3:** Statistical results for the logistic regression and the linear regression analyses for each validated metabolite.

Analysis	Statistical estimates	Adenine	Guanine	Phenyl sulfate	Citrulline	Ecgonine	Pregnenolone sulfate
R1	p-value	0.3391	**0.0038**	0.3167	0.2556	0.3099	**0.0074**
	OR [CI95]	1.08 [0.92, 1.27]	0.76 [0.63, 0.92]	1.09 [0.92, 1.28]	1.10 [0.93, 1.29]	1.09 [0.92, 1.28]	1.27 [1.06, 1.51]
R2	p-value	0.4232	**0.0158**	0.2747	0.8221	0.6235	0.1237
	OR [CI95]	1.08 [0.89, 1.30]	0.77 [0.61, 0.95]	1.12 [0.91, 1.36]	1.02 [0.84, 1.24]	1.05 [0.86, 1.28]	1.18 [0.95, 1.46
R4	p-value	0.7191	**0.0005**	0.5026	0.3718	0.9690	**0.0032**
	OR [CI95]	1.03 [0.86, 1.24]	0.69 [0.57, 0.85]	1.07 [0.88, 1.29]	1.09 [0.91, 1.30]	1.00 [0.83, 1.20]	1.34 [1.10, 1.63]
R3	p-value	0.63704	**0.00001**	0.31795	0.13706	0.20082	**0.00370**
	Beta	0.018	-0.162	0.038	0.058	-0.048	0.107
R5	p-value	0.95265	0.05785	0.34389	**0.01987**	0.91569	0.05980
	Beta	-0.003	-0.103	0.053	0.132	-0.006	0.102
R6	p-value	0.41235	**0.00004**	0.53116	0.94619	0.14473	**0.01171**
	Beta	0.044	-0.213	0.033	-0.004	-0.076	0.128

CI, confidence interval; OR, odds ratio. Bold values indicate significance.

**Figure 3:**
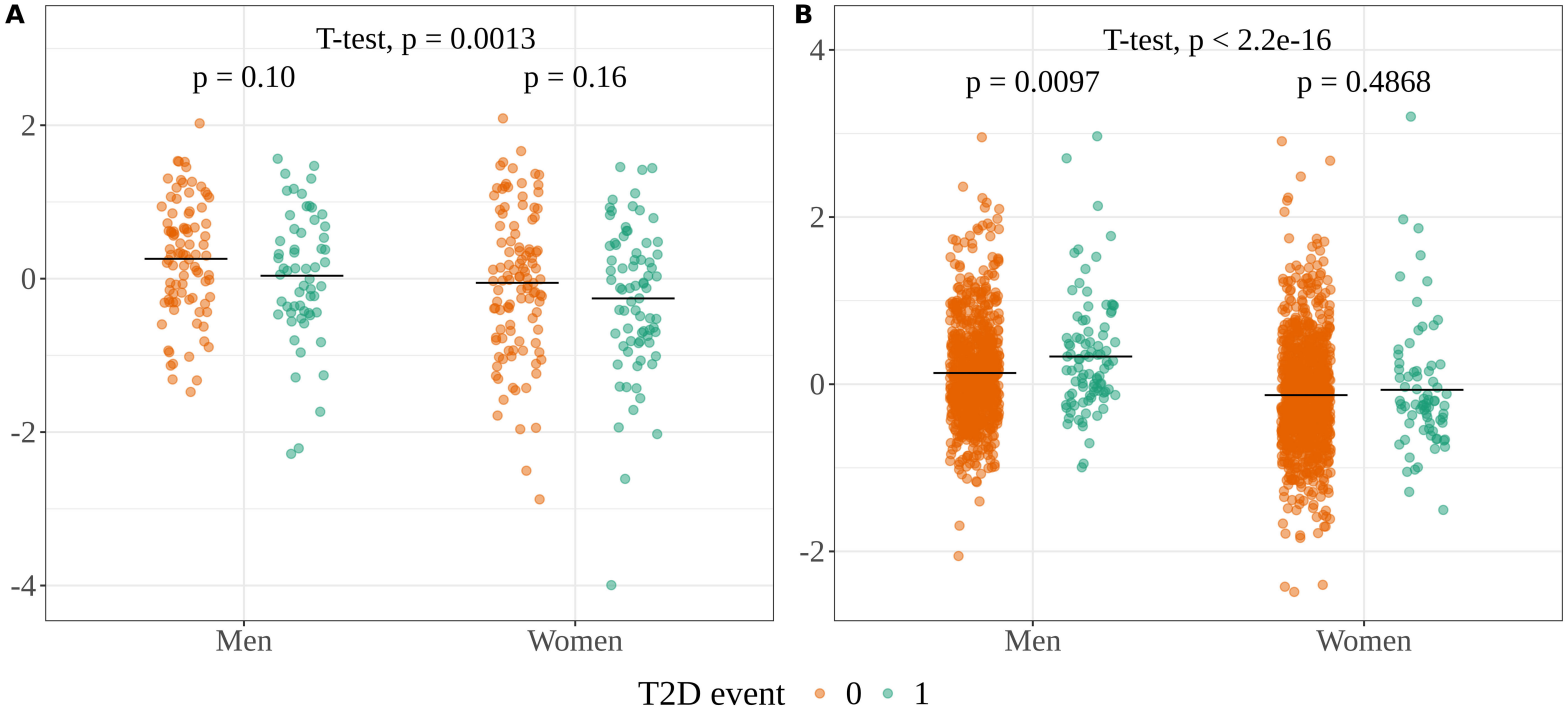
Sex-specific boxplots of the residuals of pregnenolone sulfate for the **(A)** discovery and **(B)** validation populations. The stat_compare_means function from ggpubr R package (50) was used to obtain the p-values shown in the plot.

Variability in pregnenolone sulfate intensities due to a set of clinical covariates used for model adjustment, except sex, was removed in both the discovery and validation cohorts by applying linear models and extracting the residuals. The covariables used were age, BMI, hypertension, insulin resistance, smoking habit, time-to-event, family history of diabetes and glucose in the discovery cohort, and age, BMI, hypertension, smoking habit, time-to-event, and prediabetes in the validation cohort. These residuals are plotted in **Figure 3**, illustrating sex-specific differences in pregnenolone sulfate levels in relation to incident T2D.

A stratified k-fold cross-validation analysis of three weighted logistic regression models across three data subsets showed that the inclusion of metabolites did not significantly improve predictive performance of incident T2D (**Supplementary Table S5**). Model M1 included only clinical variables (sex, age, BMI, hypertension, and smoking), with baseline prediabetes status added when analyzing all subjects or excluding those with follow-up prediabetes. Model M2 added all six validated metabolites to the clinical variables, whereas Model M3 included only the clinically significant metabolites, guanine, and pregnenolone sulfate.

## Discussion

Using an untargeted metabolomics approach, we identified pathways significantly altered in incident T2D, investigated potential metabolic biomarkers for T2D prediction in a discovery cohort, and validated six of these metabolites in an independent population through several analyses. The association with incident diabetes or glycemia was confirmed in three of the metabolites in the validation cohort. To the best of our knowledge, this is the first study to reveal significant associations between guanine, pregnenolone sulfate, and citrulline with incident T2D or glycemic transition in two independent cohorts.

In the discovery phase, technical bias was addressed using three different methods, resulting in different sets of significant features and highlighting the importance of correction procedures. While further analysis is needed, normalization using common principal components showed strong potential, as metabolites consistently significant in both populations were identified through CPCA in the discovery cohort.

Our findings revealed a broad panel of metabolites associated with T2D incidence; however, only six could be identified with high confidence. While this is common in metabolomics, in order to extend the insights gained from the discovery cohort, we conducted pathway enrichment analysis with FELLA (27) using putative annotations obtained via mWISE (8), following a similar strategy to the widely used mummichog method (7).

The following section discusses potential biological mechanisms underlying the observed associations. However, in this type of metabolomics study, the specific relationship between the alterations detected and the variable under investigation cannot be fully elucidated, and thus causality cannot be confirmed. Therefore, we report correlations between the observed associations and the studied conditions and propose potential mechanisms that may be involved.

Purine and nucleotide metabolism, two closely related pathways, were significantly altered across analyses. Nucleotide metabolism has been repeatedly found to be associated with T2D, with elevated ATP and its derivatives promoting insulin resistance (29). This is consistent with the increased adenine levels observed in our discovery cohort (**Figure 2**) and its consistent annotation via mWISE (**Supplementary Figures S9**, **S10**). Elevated ATP is also linked to platelet hyperactivity and vascular complications (30), whereas endogenous adenine may drive kidney disease in diabetic models (31). These findings suggest that such metabolic alterations may precede diabetes onset. Conversely, cyclic GMP is associated with improved mitochondrial function and insulin sensitivity (29), which may relate to the observed reductions in its precursors, guanine and guanosine. Guanine was significantly decreased in both cohorts with high-confidence identification, and both metabolites were annotated by mWISE as significantly reduced (**Supplementary Figures S9**, **S10**). This decline may reflect oxidative degradation, as elevated 8-hydroxyguanine, a guanine oxidation product, has been reported in T2D (32). In addition, uridine, which regulates glycogen synthesis, has been reported to be elevated in individuals with T2D (29), and this trend was also observed in our data (**Supplementary Figure S10**). These findings support a role for disrupted purine and pyrimidine metabolism in T2D risk.

Another group of interconnected pathways significantly altered in association with incident T2D across multiple analyses includes glycolysis and gluconeogenesis, galactose metabolism, and fructose and mannose metabolism. These carbohydrate metabolism pathways are central to glucose homeostasis, so their alteration is consistent with the metabolic disturbances underlying T2D.

We also found caffeine metabolism significantly altered, with all identified metabolites, including caffeine itself, positively associated with T2D incidence (**Supplementary Figure S11**). Prior studies have demonstrated that caffeine intake is associated with increased postprandial plasma glucose and insulin in individuals with type 2 diabetes (33) and with reduced insulin sensitivity in healthy adults (34). This raises concerns that habitual caffeine consumption could contribute to a higher diabetes risk (33). Elevated epinephrine levels induced by caffeine have been proposed to explain this relationship, as epinephrine is known to stimulate hepatic glucose production and inhibit glucose uptake in muscle and adipose tissue (34). The results of our study point to a caffeine-associated increase in the risk of developing type 2 diabetes.

The ATP-binding cassette (ABC) transporter pathway was significantly altered in two of four analyses and has been widely linked to T2D, particularly its macrovascular complications. T2D promotes atherosclerosis, partly through diabetic dyslipidemia. We previously reported disrupted lipid metabolism in T2D patients with subclinical carotid atherosclerosis (35). High-density lipoprotein (HDL) cholesterol, protective against cardiovascular disease, facilitates reverse cholesterol transport via ABC transporters ABCA1 and ABCG1. In T2D, ABCA1-mediated cholesterol efflux is impaired in small HDL particles (36), and hepatic ABCA1 deficiency in mice reduces β-cell function and glucose tolerance (37). Our results support a role for altered ABC transporter activity in T2D pathogenesis.

Pregnenolone sulfate, a steroid biosynthesis metabolite, was significantly associated with incident T2D or glycemia status in both cohorts. Interestingly, this was the only metabolite showing opposite trends: decreased in the discovery cohort but increased in the validation cohort, a discrepancy also reported in previous studies, where pregnenolone sulfate was found either elevated or reduced in T2D (38–40). Elevated levels of pregnenolone may represent a counter-regulatory protective mechanism (38), with levels varying by disease stage. The two cohorts differed notably in time-to-T2D onset (mean 7.4 *vs*. 3.1 years), potentially explaining this inversion. To further investigate this inconsistency and given prior evidence from our lab on sex-specific lipid metabolism in diabetes (41), we explored sex differences (**Figure 3**) and found that only men contributed to the significant increase in pregnenolone sulfate in the validation cohort. This aligns with findings of reduced testosterone and elevated dehydroepiandrosterone sulfate (DHEA-S) levels in men with early T2D (42), and the biochemical relationship between DHEA-S and pregnenolone sulfate formation (43). These results suggest that elevated pregnenolone sulfate levels in men may reflect early-onset diabetes in the validation cohort, although further sex-specific investigation is needed.

Other validated metabolites not involved in the discussed pathways include ecgonine, phenyl sulfate, and citrulline. Ecgonine, a cocaine metabolite, was significantly reduced in future T2D cases; however, the reason for this association remains unclear. Notably, a related compound was previously reduced in placentas from gestational diabetes cases (44). Phenyl sulfate, which has been linked to albuminuria (45) and diabetic kidney disease (46), was significantly elevated in subjects who developed T2D in one analysis. Citrulline was positively associated with T2D development in multiple analyses. It was also linked to glycemia status and transitions in the validation cohort. Citrulline, a non-proteinogenic amino acid, participates in the citrulline–nitric oxide (NO) cycle, where it is converted to arginine by argininosuccinate synthase (ASS) and argininosuccinate lyase (ASL), and then back to citrulline by nitric oxide synthase (NOS), releasing NO (47). Reduced endothelial NO production is a hallmark of diabetes pathogenesis. Insulin normally maintains NO synthesis by upregulating endothelial NOS and ASS (48). Thus, increased citrulline in our populations could be explained by the downregulation of ASS caused by the initial states of insulin deficiency or impaired insulin action in T2D incidence.

Although several metabolites showed significant associations with incident T2D in two independent cohorts, they did not demonstrate predictive power in the validation cohort. This may be partly explained by the limited number of incident T2D cases in that cohort. In addition, heterogeneity between the discovery and validation populations may have influenced predictive performance, particularly differences in disease stage. The shorter time-to-event found in the validation cohort suggests a more advanced progression stage from prediabetes to T2D compared with the discovery cohort.

Our study has several strengths. First, models were adjusted for multiple confounders, with the discovery cohort matched by sex, age, BMI, and time-to-event. Second, the untargeted metabolomics approach enabled a comprehensive analysis, and identification using standards ensured high confidence. Most importantly, findings were validated in a large, independent cohort. However, there are also some limitations, such as the imbalance of incident T2D cases in the validation cohort and the differences between the two populations. These factors may explain why, despite that three out of the six metabolites (guanine, pregnenolone sulfate, and citrulline) maintain statistical significance, they lack predictive power. Prior work using electronic health records have demonstrated differential trajectories in subjects with T2D (49), suggesting that integrating such insights and increasing sample sizes in discovery could improve biomarker identification. Larger studies are needed to capture physiological diversity and better predict T2D progression.

In this study, we identified and validated six metabolites and discovered several altered pathways associated with incident type 2 diabetes (T2D) using an untargeted metabolomics approach. Metabolites such as guanine, pregnenolone sulfate, and citrulline showed potential as biomarkers for T2D progression. Despite rigorous bias correction and validation, their predictive power was limited in the validation cohort, possibly due to cohort differences. Still, our findings lay the groundwork for future research into reliable T2D biomarkers, emphasizing the need for larger, more diverse populations and consideration of sex-specific and longitudinal factors.

## Data Availability

The original contributions presented in the study are included in the article/**Supplementary Material**. Further inquiries can be directed to the corresponding author/s.
