# Supplementary material for: Guanine and pregnenolone sulfate are associated with incident type 2 diabetes in two independent populations
Source: Front Endocrinol (Lausanne). 2025 Dec 3;16:1706886. doi: 10.3389/fendo.2025.1706886 (PMC12708234; doi:10.3389/fendo.2025.1706886)
Supplement: Supplementary file 1 [file DataSheet1.pdf]

Supplemental Material - Guanine and pregnenolone sulfate are associated with incident type 2 diabetes in two independent populations

Maria Barranco-Altirriba[1,2,3,4], Minerva Granado-Casas[5,6,7,8], Oscar Yanes[5,9], Jordi Capellades[5,10], Alexandra Junza[5,11], Josep Franch-Nadal[5,6], Joan Vendrell[5,12], Gemma Llauradó[5,13], Sergio Valdés[5,14], Eva García-Escobar[5,14], Marcelino Bermúdez-López[15,16], José Manuel Valdivielso[16], Victor-Miguel López-Lifante[17,18,19], Cecilia Herrero-Alonso[18], Mireia Falguera[6,20,], Maria Belén Vilanova[6,21], Ingrid Arteaga[18,22], Pere Torán-Monserrat[18,23,24], Alexandre Perera-Lluna[2,3,4], Esmeralda Castelblanco[25*], Didac Mauricio[1,5,26,27*]

1. Department of Endocrinology & Nutrition, Hospital de la Santa Creu i Sant Pau, Barcelona, Spain.

2. B2SLab, Department of Systems Engineering, Automatics, and Industrial Informatics, Universitat Politècnica de Catalunya, Barcelona, Spain.

3. Networking Biomedical Research Centre in the subject area of Bioengineering, Biomaterials and Nanomedicine (CIBER-BBN), Instituto de Salud Carlos III (ISCIII), Madrid, Spain.

4. Institut de Recerca Sant Joan de Déu, Esplugues de Llobregat, Barcelona, Spain.

5. CIBER of Diabetes and Associated Metabolic Diseases (CIBERDEM), Instituto de Salud Carlos III (ISCIII), Barcelona, Spain

6. DAP-Cat Group, Research Support Unit, Institut Universitari d'Investigació en Atenció Primària Jordi Gol, Barcelona, Spain.

7. GESEC group, Department of Nursing and Physiotherapy, Faculty of Nursing and Physiotherapy, University of Lleida, Montserrat Roig, 25198 Lleida, Spain

8. Healthcare Research Group (GRECS), Institute of Biomedical Research in Lleida (IRBLleida), Av. Alcalde Rovira Roure, 80, 25198 Lleida, Spain

9. Universitat Rovira i Virgili, Department of Electronic Engineering, IISPV, Tarragona, Spain.

10. Institute of Health Research Pere Virgili (IISPV), Tarragona, Spain.

11. Scientific and Technical Resources Services, Universitat Rovira i Virgili, Tarragona (Spain).

12. Department of Endocrinology and Nutrition, Research Unit, Institut d'Investigació Sanitària Pere Virgili (IISPV) - Hospital Universitari de Tarragona Joan XXIII, Universitat Rovira i Virgili, Tarragona, Spain

13. Department of Endocrinology and Nutrition, Hospital del Mar, Hospital del Mar Medical Research Institute (IMIM), Barcelona, Spain

14. UGC Endocrinología y Nutrición. Hospital Regional Universitario de Málaga. IBIMA-Plataforma BIONAND, Málaga, Spain

15. Experimental Medicine department, University of Lleida, Lleida, Spain.

16. Vascular & Renal Translational Research group, Institut de Recerca Biomédica IRBLleida, Lleida, Spain

17. Faculty of Medicine, Universitat Autònoma de Barcelona (UAB), Barcelona, Spain.

18. Unitat de Suport a la Recerca Metropolitana Nord, Institut Universitari d'Investigació en Atenció Primària Jordi Gol (IDIAP Jordi Gol), Mataró, Spain

19. Primary Healthcare Palau-solità i Plegamans, Gerència d'Àmbit d'Atenció Primària Metropolitana Nord, Institut Català de la Salut, 08186 Lliçà d'Amunt, Barcelona, Spain

20. Institute for Biomedical Research Dr. Pifarré Foundation IRB Lleida, University of Lleida and Primary Health Care Centre Tàrrega, Gerència d'Atenció Primaria, Institut Català de la Salut, Lleida, Spain.

21. Primary Health Care Centre Igualada Nord, Consorci Sanitari de l'Anoia, Igualada, Spain.

22. Primary Healthcare Center Vall del Tenes, Gerència d'Àmbit d'Atenció Primària Metropolitana Nord, Institut Català de la Salut, 08186 Lliçà d'Amunt, Barcelona, Spain

23. Department of Medicine, Faculty of Medicine, Universitat de Girona, Girona, Spain

24. Multidisciplinary Research Group in Health and Society (GREMSAS), Institut Universitari d'Investigació en Atenció Primària Jordi Gol (IDIAPJGol), Barcelona, Spain

25. Division of Endocrinology, Metabolism and Lipid Research, Department of Medicine, Washington University School of Medicine, St. Louis, MO 63110, USA.

26. Institut d'Investigació Biomèdica Sant Pau (IR-Sant Pau), 08041, Barcelona, Spain.

27. Faculty of Medicine, University of Vic - Central University of Catalonia, Vic, Spain.

* Correspondence: didacmauricio@gmail.com (D.M.); esmeralda@wustl.edu (E.C.); Tel.: +34-935-565-661 (D.M.); +1-314-747-0437 (E.C.); Fax: +34-935-565-602 (D.M.)

Research Design and Methods

Study Populations

Discovery

In the Di@bet.es study, 5,072 subjects older than 18 years old were randomly selected from the National Health System registries in Spain from 100 primary health care centers. Subjects with diabetes at baseline, serious illness, pregnancy, recent delivery or lactation, or surgery within the previous month were excluded. A subset of subjects was unable or unwilling to participate at follow-up, yielding a final sample of 2,408 subjects completed the study.

Socio-demographic and clinical data, survey on habits, anthropometric data and blood pressure were acquired. Blood samples were collected in the fasting state and an oral glucose tolerance test was performed. Determinations of serum glucose were made. At follow-up, the same variables were collected and HbA1c was determined.

Validation

The validation data was built using four cohorts from Catalonia (Figure S1):

The ILERVAS(1,2) project was a population-based study focused on subclinical arterial disease and hidden kidney disease. The study enrolled a total of 8,330 subjects from 2015 to 2018 fulfilling the inclusion criteria: women aged between 50 and 70; men between the ages of 45 and 65; presence of at least one CV risk factor; no prior history of: CV disease, diabetes, CKD or active neoplasm; and a life expectancy longer than 18 months. Sociodemographic variables, medical history of comorbidities and medical treatments were collected from the electronic Primary Care health care records. Vascular diagnostic tests, spirometry, anthropometric and clinical-chemistry data were done in a mobile unit. Physical activity was assessed using the International Physical Activity Questionnaire (IPAQ), diet data using the survey adapted from the PREDIMED study, and information on sleep pattern using the Berlin questionnaire and the Epworth somnolence scale.

The prospective Mollerussa cohort study(3) was a population-based study conducted in a semirural area of Catalonia (Spain). From 24,666 subjects with registered health records that met the inclusion criteria (i.e., subjects older than 25 years and attending the same health area), 2226 individuals were randomly

selected and invited to participate. Subjects with a previous diagnosis of any type of diabetes or on treatment with oral antidiabetic drugs were excluded. Finally, a total number of 594 subjects willing to participate and with baseline laboratory data remained after applying the exclusion criteria. Sociodemographic variables, education level, sector of working activity, self-perceived work activity, physical activity obtained using the Spanish-validated IPAQ, family history of hypertension, dyslipidemia, hypertriglyceridemia, diabetes mellitus and acute myocardial infarction or angina pectoris, personal history of hypertension, dyslipidemia, hypertriglyceridemia, alcohol consumption and smoking habit, current medication, anthropometric measures and physical examination, nutritional data, intima-medial thickness measured through carotid ultrasound imaging and complete blood count, lipid, liver, kidney and thyroid profile, FPG and HbA1c were acquired.

FIBROSCAN(4) is a population-based cross-sectional study aimed at determining the prevalence of liver fibrosis among subjects recruited between 2012 and 2016 in an urban area of Catalonia (Spain). Participants were randomly selected from a total of 162,950 subjects aged 18 to 75 years. Subjects with a current history of liver disease, active malignancy, other severe diseases, or admitted to long-term nursing homes were excluded. A total of 3,076 subjects participated in the study, from which 52 were excluded, resulting in a final population of 3014 subjects. A detailed medical history, anthropometric measurements, blood tests (including glycemia and glycosylated hemoglobin), and a transient elastography were acquired.

ARTPER(5) is a cross-sectional and multicenter population-based study aimed at identifying the prevalence of symptomatic and asymptomatic peripheral arterial disease and related factors in a population of Catalonia, Spain. Individuals were randomly selected and invited to participate. A total of 3786 subjects older than 49 years were recruited from 2006 to 2008. Demographic, cultural and economic variables, personal history of hypercholesterolemia, diabetes mellitus, arterial hypertension and smoking habits, anthropometric measurements, systolic/diastolic blood pressure and blood analysis were acquired. After 5 years of follow-up, subjects were revisited. We selected patients from the second stage of the study, younger than 71 years old, with blood sample and without prevalent cardiovascular disease.

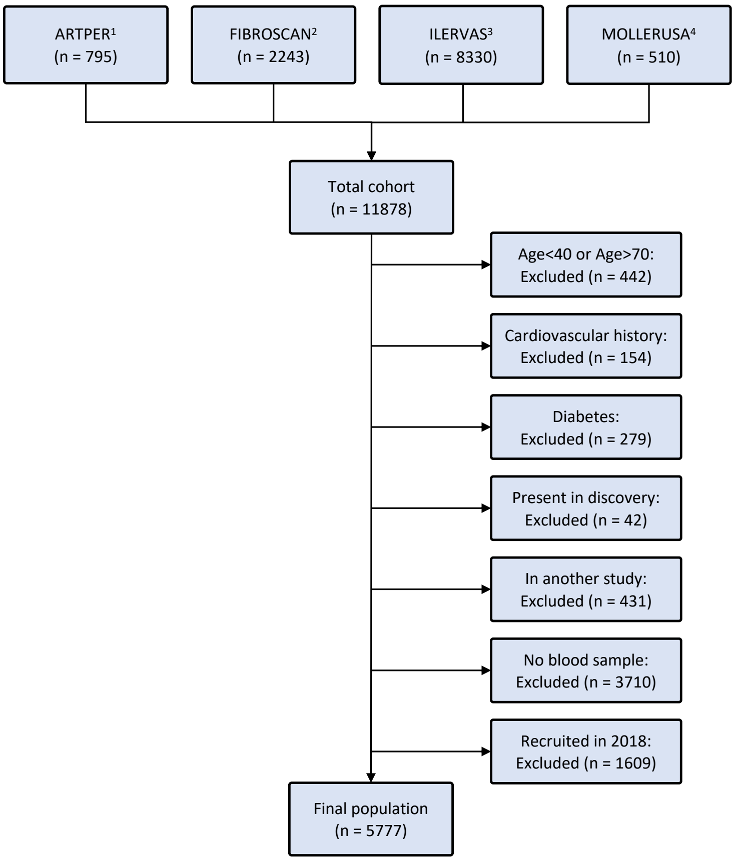

Figure S1. Workflow for the selection of participants in the validation cohort. The boxes show the number of participants that fulfill each criterium. One participant can fulfill more than one criterium.

Targeted analysis

Table S1. m/z and polarity used to determine the metabolites by LC-MS.

Metabolite	*m/z*	Polarity
Guanine	152.0567	Positive
Adenine	134.0472	Negative
Citrulline	174.0884	Negative
Pregnenolone sulfate	395.1898	Negative
Phenyl sulfate	172.9914	Negative

Statistical analysis – discovery

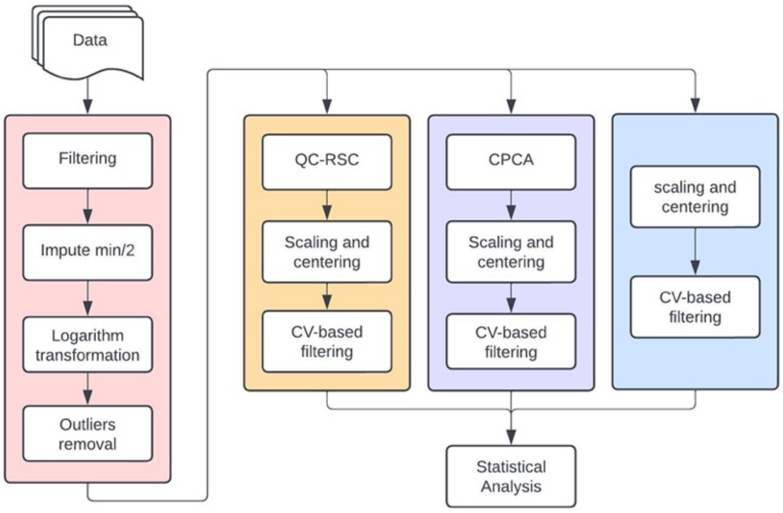

Figure S2. Workflow for data preparation. From the intensity peak list to the statistical analysis.

Table S2. Statistical analyses performed.

Code	Variable of interest	Model	Subjects
R1	T2D development	Logistic regression	All
R2	T2D development	Logistic regression	Prediabetes at t_0
R3	Glycemia status	Linear regression	All
R4	T2D development	Logistic regression	No prediabetes at t_f
R5	Glycemia transition	Linear regression	Normoglycemia at t_0
R6	Glycemia transition	Linear regression	Prediabetes at t_0

t_0=baseline; t_f=follow-up

Results

Table S3. Number of LC-MS features before and after filtering.

Correction	Ionization	Initial	After 80%	After CV filter
None	Positive	2545	2297	2128
	Negative	2187	2063	1734
CPCA	Positive	2545	2297	2099
	Negative	2187	2063	1719
QC-RSC	Positive	2545	2297	2137
	Negative	2187	2063	1785

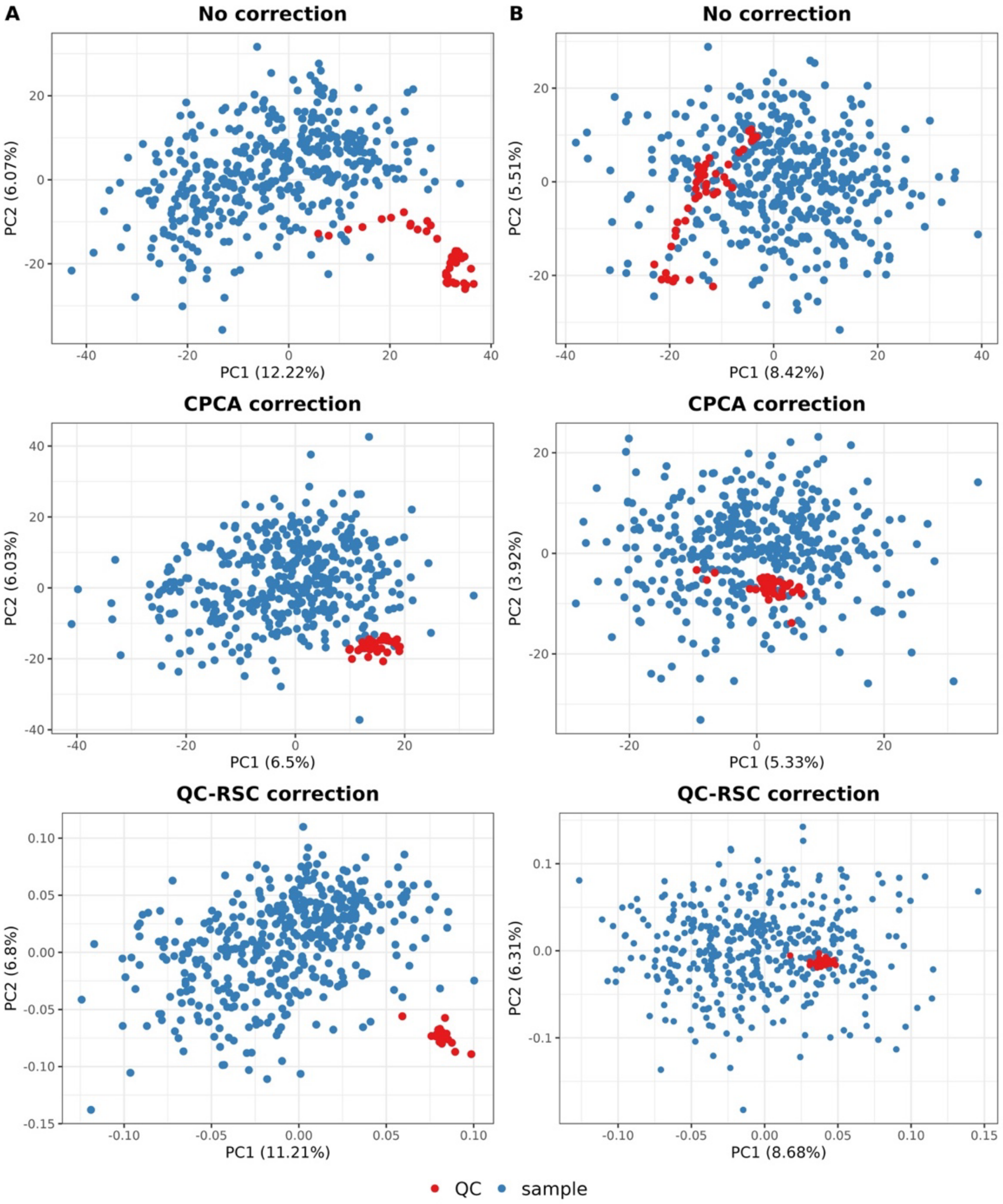

Figure S3. Principal component analysis (PCA) plots of the three paths used to treat technical bias: without QC-based correction, using common principal component analysis (CPCA)-based correction and Quality Control-Robust Spline Correction (QC-RSC). The A column shows the positive ionization mode data and column B the negative one.

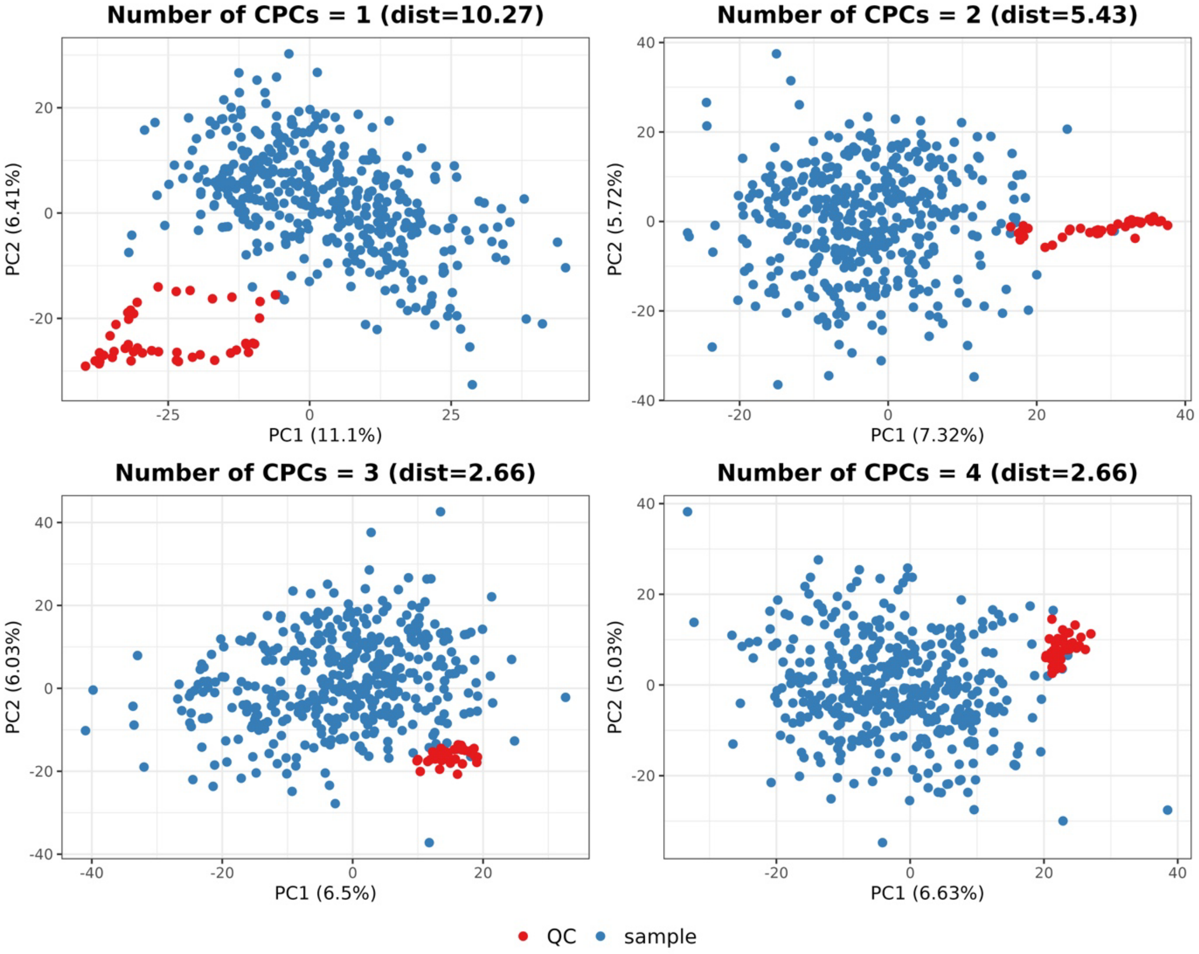

Figure S4. PCA plots of the common principal component analysis (CPCA)-based correction using one, two, three and four common principal components (CPC) for the positive ionization mode data. The plot title indicates the number of CPCs used and the euclidean distance between the QC samples.

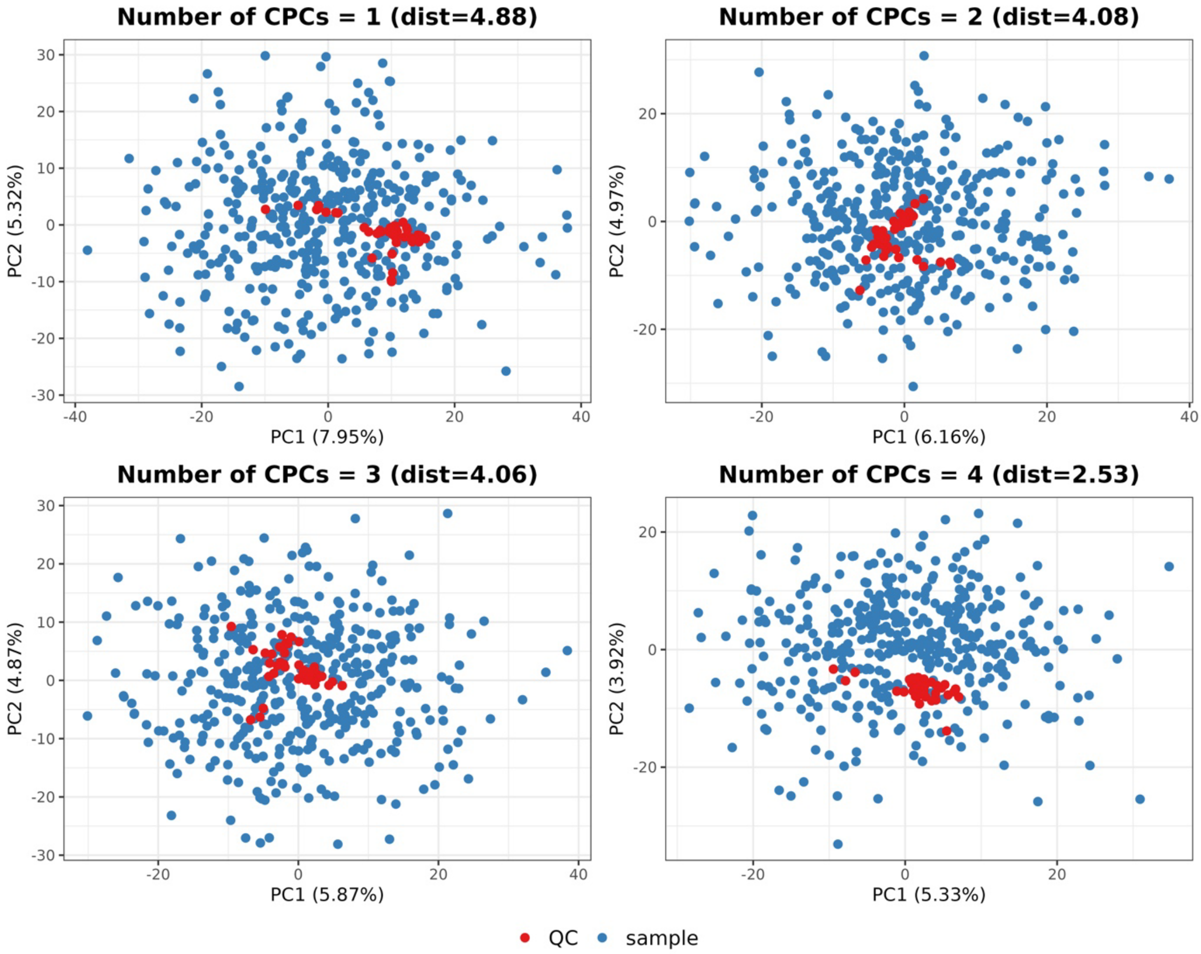

Figure S5. PCA plots of the common principal component analysis (CPCA)-based correction using one, two, three and four common principal components (CPC) for the negative ionization mode data. The plot title indicates the number of CPCs used and the euclidean distance between the QC samples.

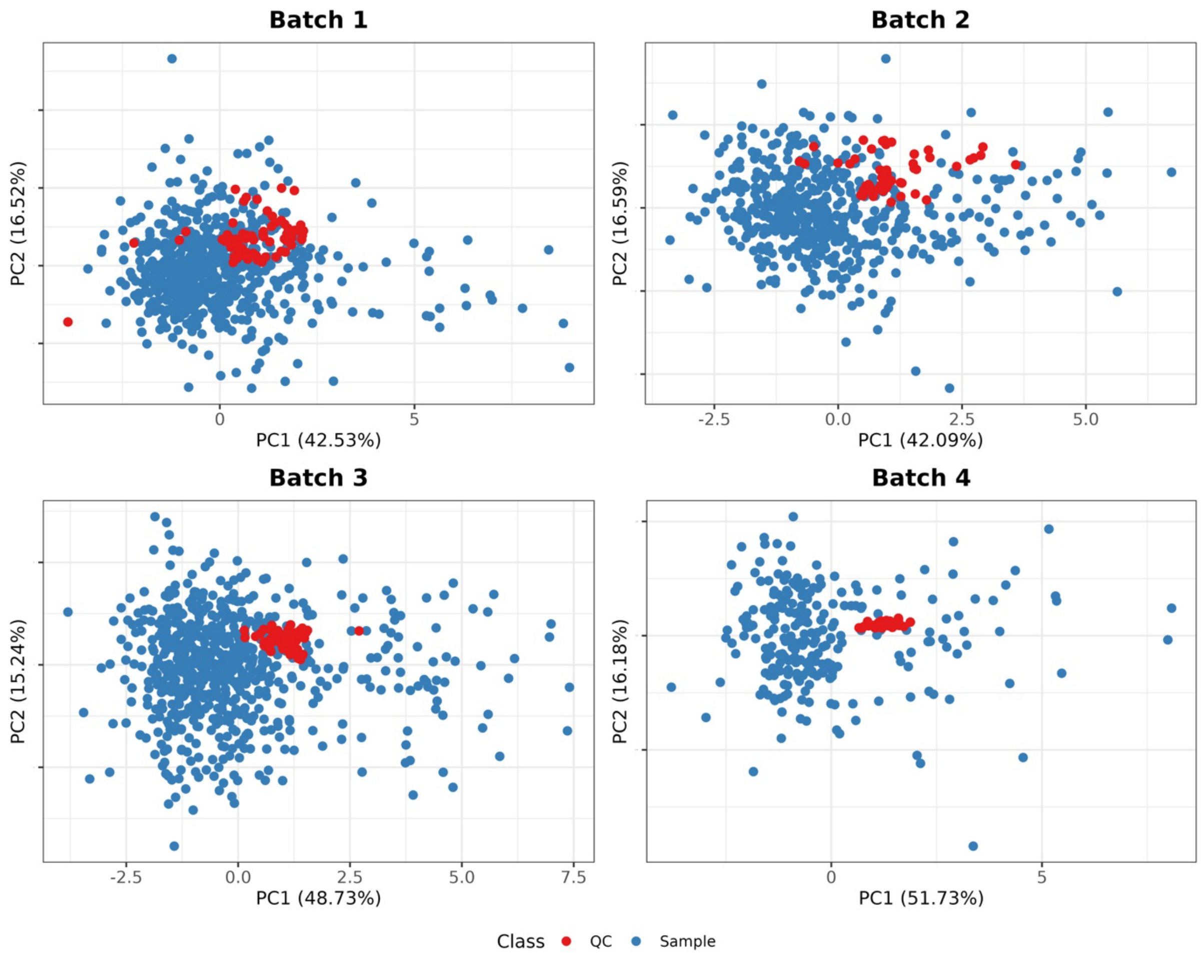

Figure S6. Visualization of technical bias in the validation cohort for each batch. The first two principal components (PCs) from the PCA are shown, with QC samples and individual samples colored differently within each batch of the validation stage.

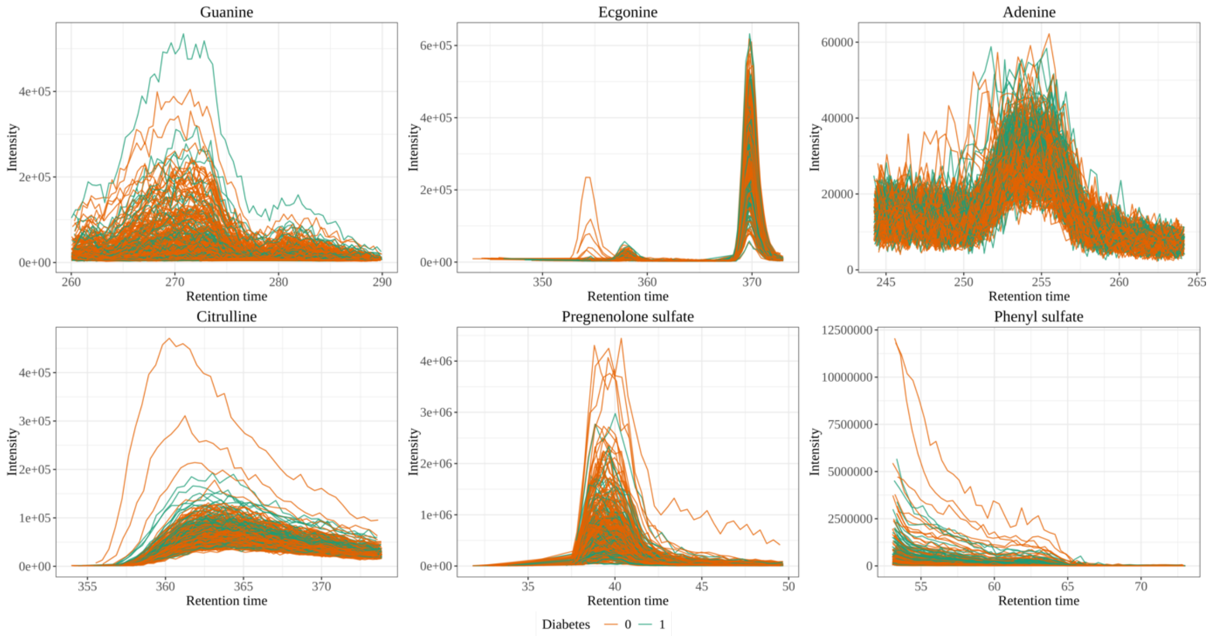

Figure S7. Chromatograms for the validated metabolites in the untargeted analysis (discovery cohort).

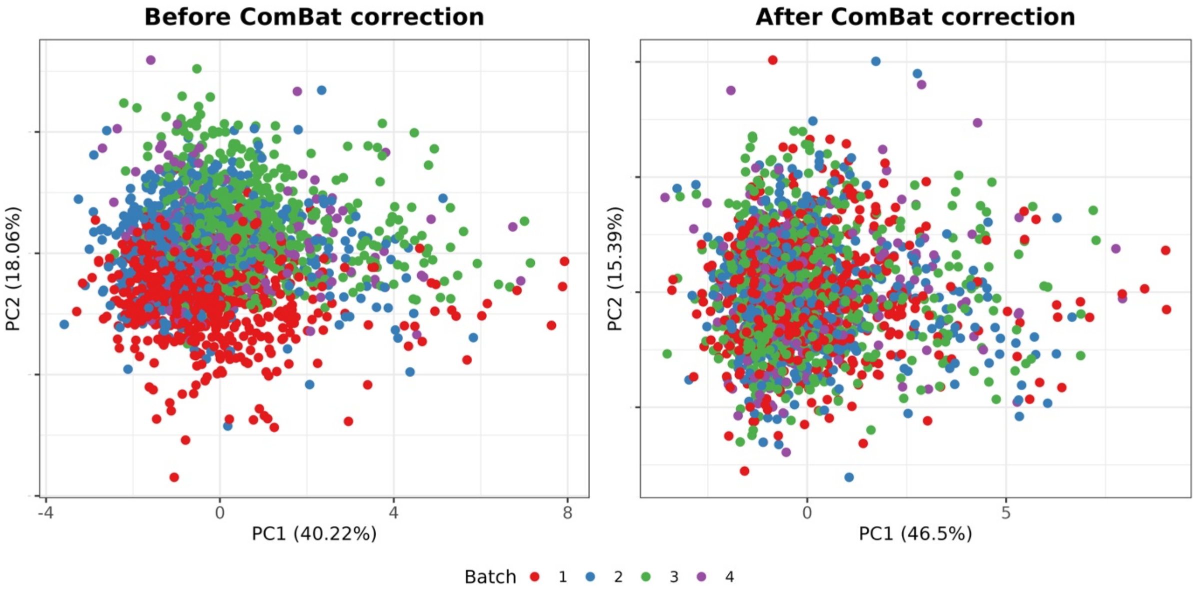

Figure S8. Visualization of batch effects in the validation cohort. The first two principal components (PCs) from the PCA are shown before and after applying ComBat batch effect correction.

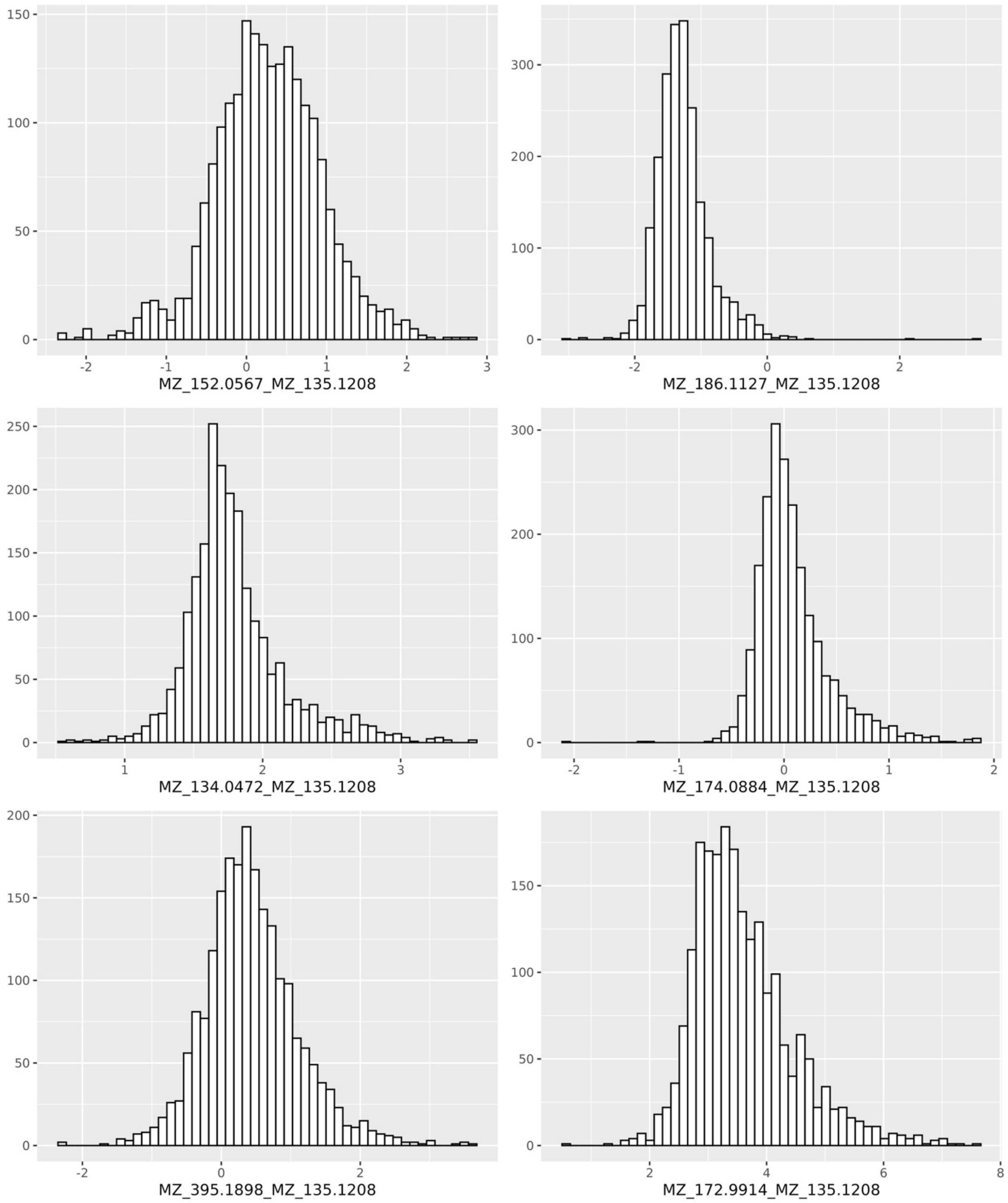

Figure S9. Histograms of the log-transformed and corrected intensity distribution of the validated metabolites.

Table S4. Number of significant features for each analysis, correction type and ionization mode.

Analysis	Ionization	CPCA	QC-RSC	None
R1	Positive	21	59	0
	Negative	120	51	11
R2	Positive	0	0	0
	Negative	0	0	0
R3	Positive	60	21	7
	Negative	115	38	69
R4	Positive	42	23	0
	Negative	39	18	84
R5	Positive	26	9	7
	Negative	3	14	1
R6	Positive	0	0	0
	Negative	1	0	0

Table S5. Cross-validated prediction metrics using a weigthed logistic regression model. The mean (sd) of each metric is shown for three models types using three different sets of data.

Data	Model	Sensitivity	Specificity	F1-score	Balanced Accuracy
All	M1	0.77 (0.05)	0.65 (0.04)	0.27 (0.02)	0.71 (0.02)
All	M2	0.74 (0.08)	0.68 (0.09)	0.28 (0.03)	0.71 (0.03)
All	M3	0.73 (0.09)	0.68 (0.09)	0.28 (0.03)	0.71 (0.04)
Only prediabetes at t_0	M1	0.60 (0.13)	0.70 (0.15)	0.34 (0.05)	0.65 (0.03)
Only prediabetes at t_0	M2	0.69 (0.12)	0.60 (0.16)	0.33 (0.04)	0.64 (0.03)
Only prediabetes at t_0	M3	0.67 (0.12)	0.64 (0.17)	0.34 (0.04)	0.65 (0.03)
No prediabetes at t_f	M1	0.78 (0.10)	0.77 (0.07)	0.53 (0.02)	0.78 (0.02)
No prediabetes at t_f	M2	0.80 (0.08)	0.77 (0.07)	0.54 (0.05)	0.78 (0.03)
No prediabetes at t_f	M3	0.81 (0.10)	0.76 (0.07)	0.53 (0.04)	0.78 (0.03)

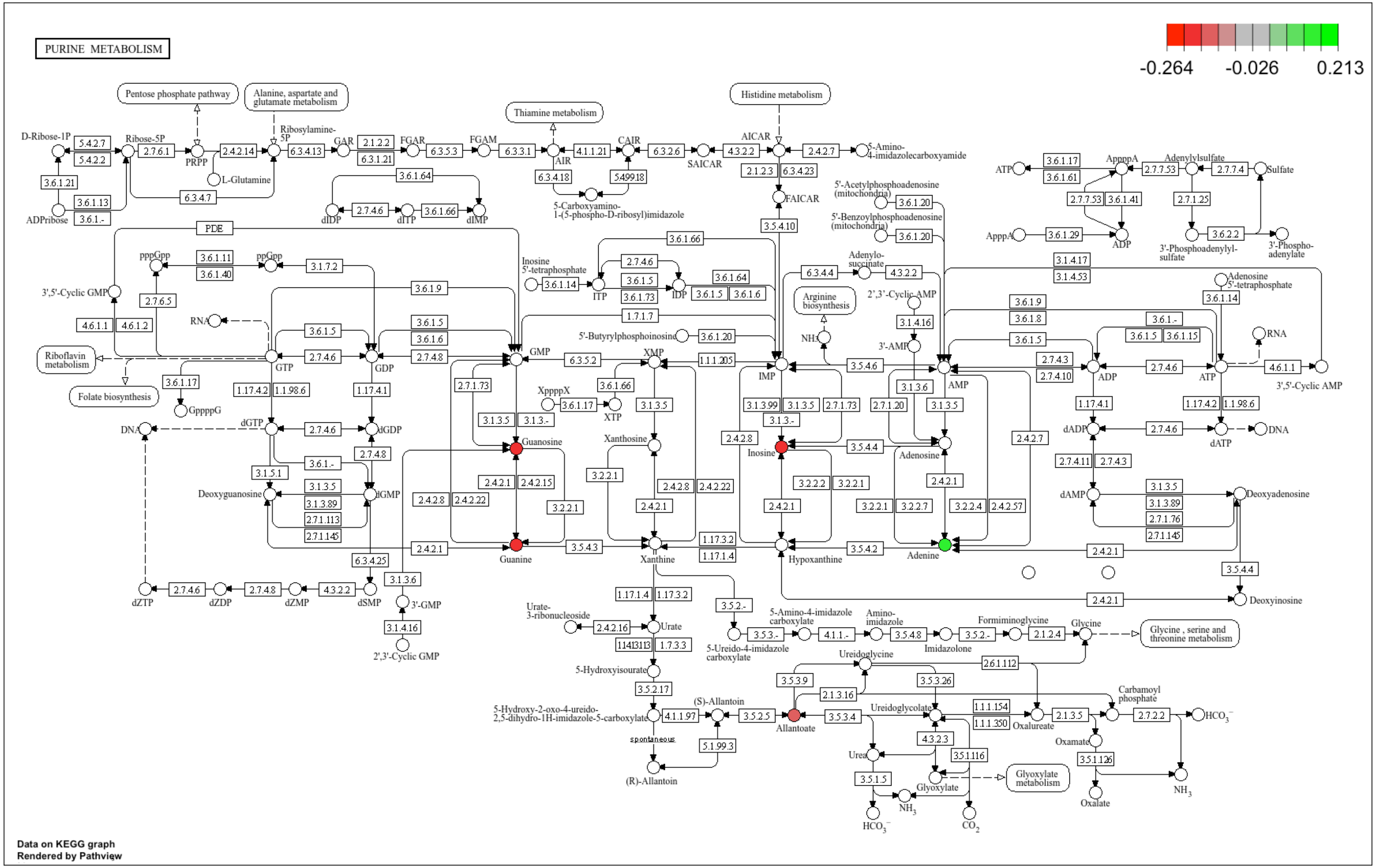

Figure S10. KEGG purine metabolism pathway map. Metabolites with significantly increased levels are highlighted in green, and those with significantly decreased levels are highlighted in red. Image generated using pathview R package (version 1.30.1).

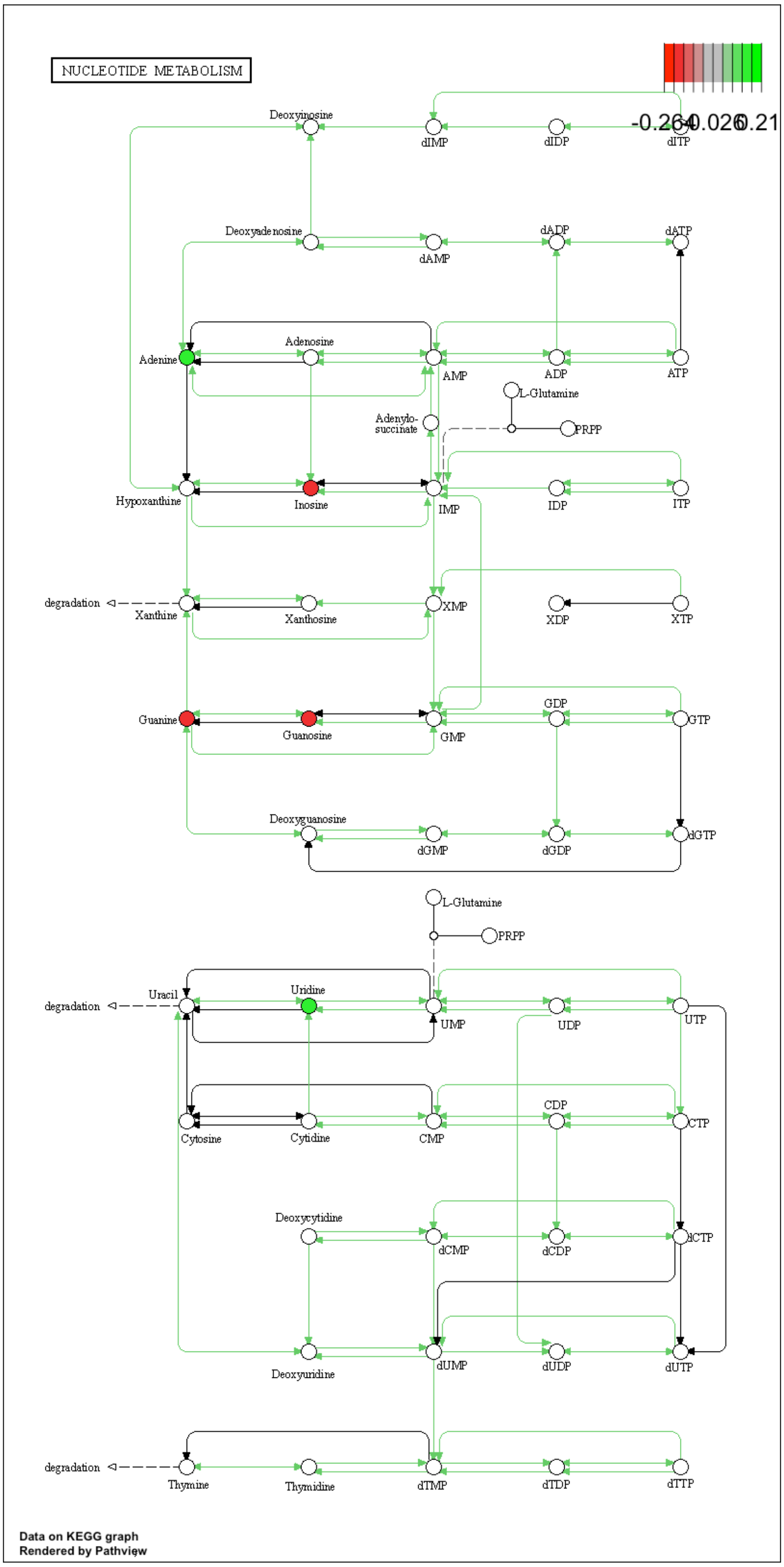

Figure S11. KEGG nucleotide metabolism pathway map. Metabolites with significantly increased levels are highlighted in green, and those with significantly decreased levels are highlighted in red. Image generated using pathview R package (version 1.30.1).

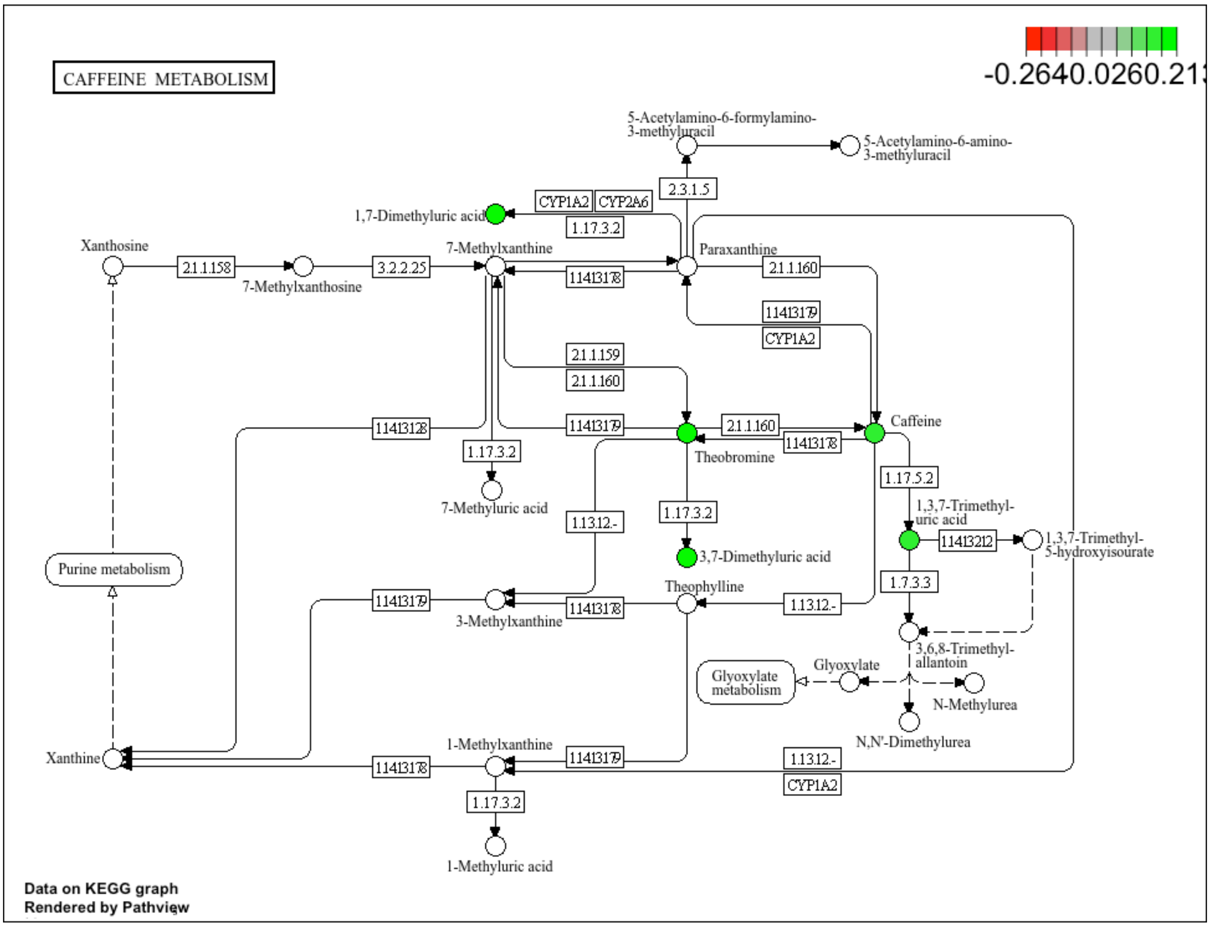

Figure S12. KEGG caffeine metabolism pathway map. Metabolites with significantly increased levels are highlighted in green. Image generated using pathview R package (version 1.30.1).

References

1. Betriu À, Farràs C, Abajo M, et al. Randomised intervention study to assess the prevalence of subclinical vascular disease and hidden kidney disease and its impact on morbidity and mortality: The ILERVAS project. *Revista de la Sociedad Española de Nefrología*. 2016;36(4):389-396.

2. Bermúdez-López M, Martínez-Alonso M, Castro-Boqué E, et al. Subclinical atheromatosis localization and burden in a low-to-moderate cardiovascular risk population: the ILERVAS study. *Rev Esp Cardiol (Engl Ed)*. 2021;74(12):1042-1053. doi:10.1016/j.rec.2021.06.008

3. Vilanova MB, Falguera M, Marsal JR, et al. Prevalence, clinical features and risk assessment of pre-diabetes in Spain: The prospective Mollerussa cohort study. *BMJ Open*. 2017;7(6). doi:10.1136/bmjopen-2016-015158

4. Caballería L, Pera G, Arteaga I, et al. High Prevalence of Liver Fibrosis Among European Adults With Unknown Liver Disease: A Population-Based Study. *Clinical Gastroenterology and Hepatology*. 2018;16(7):1138-1145.e5. doi:10.1016/j.cgh.2017.12.048

5. Alzamora MT, Forés R, Miguel Baena-Díez J, et al. The Peripheral Arterial disease study (PERART/ ARTPER): prevalence and risk factors in the general population. *BMC Public Health volume*. 2010;10(38). http://www.biomedcentral.com/1471-2458/10/38
